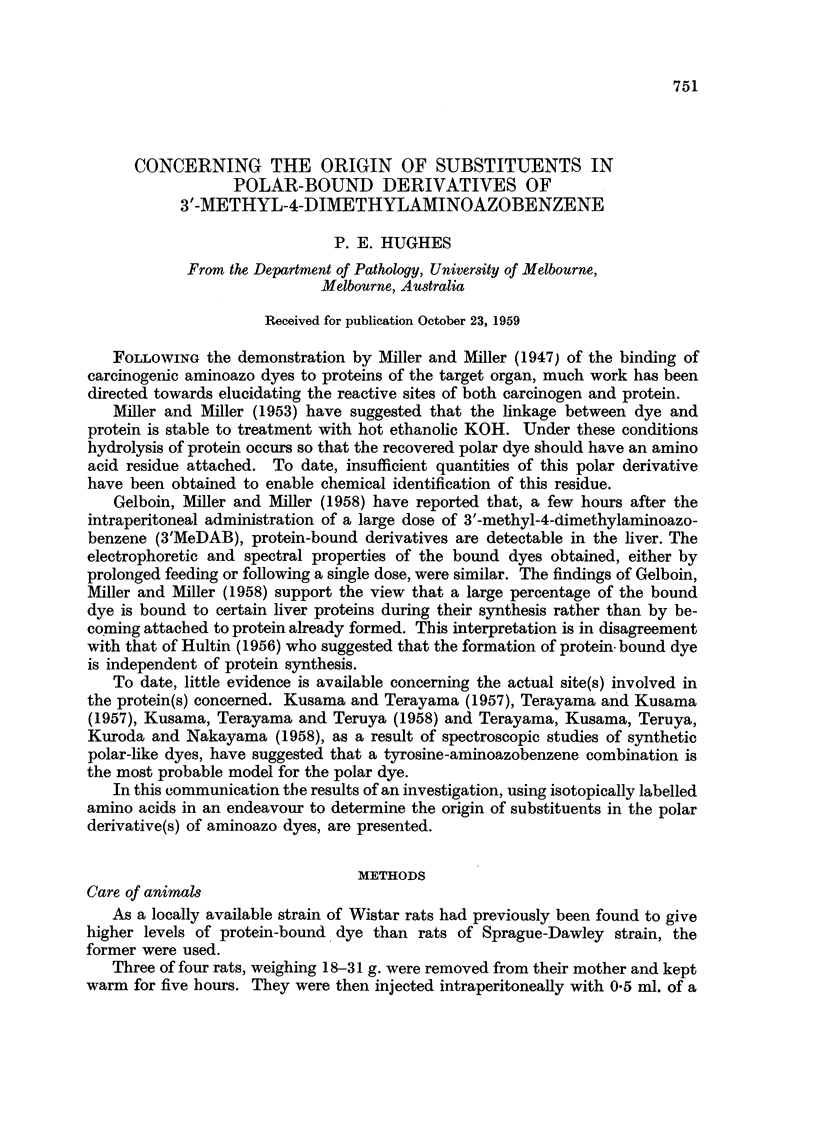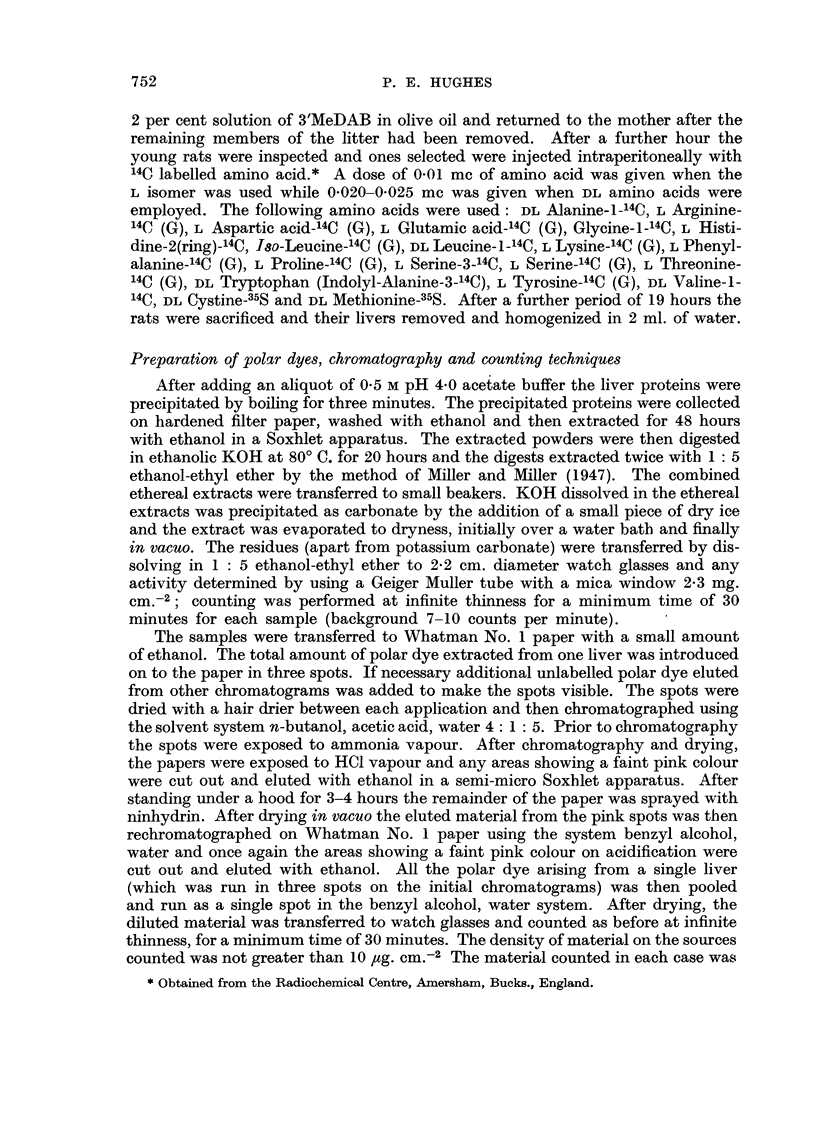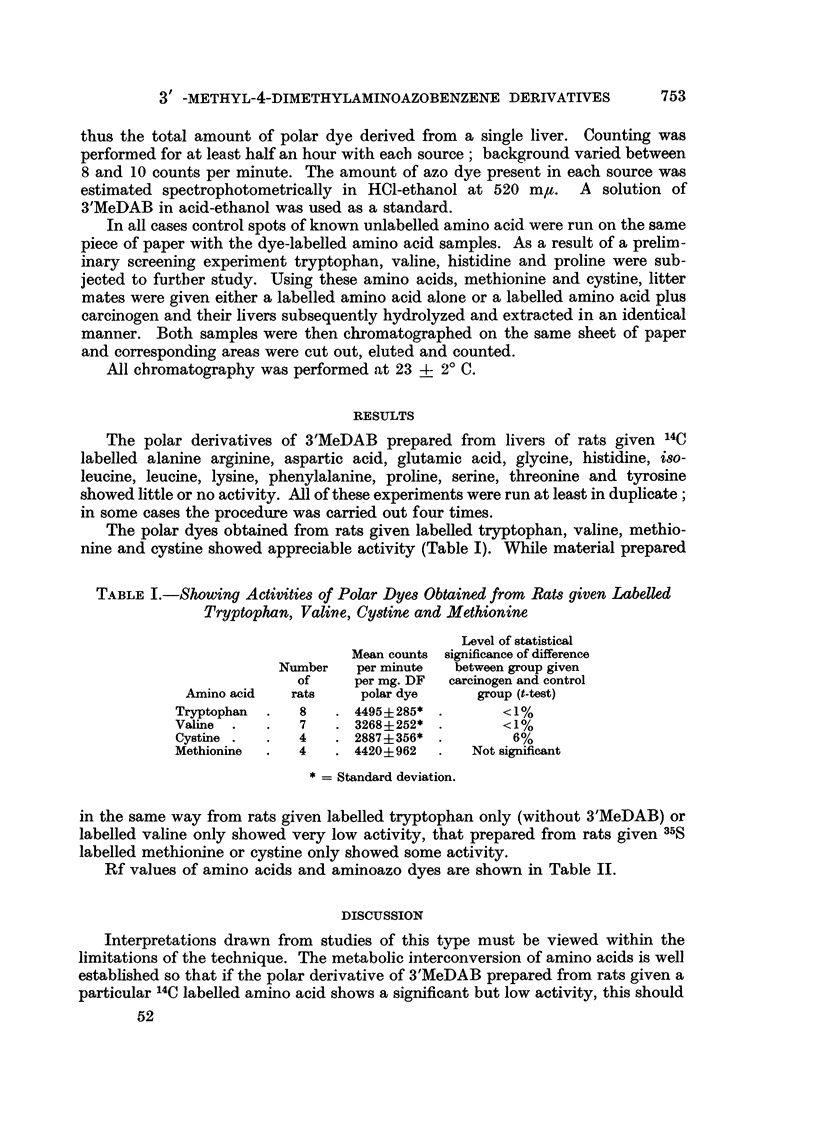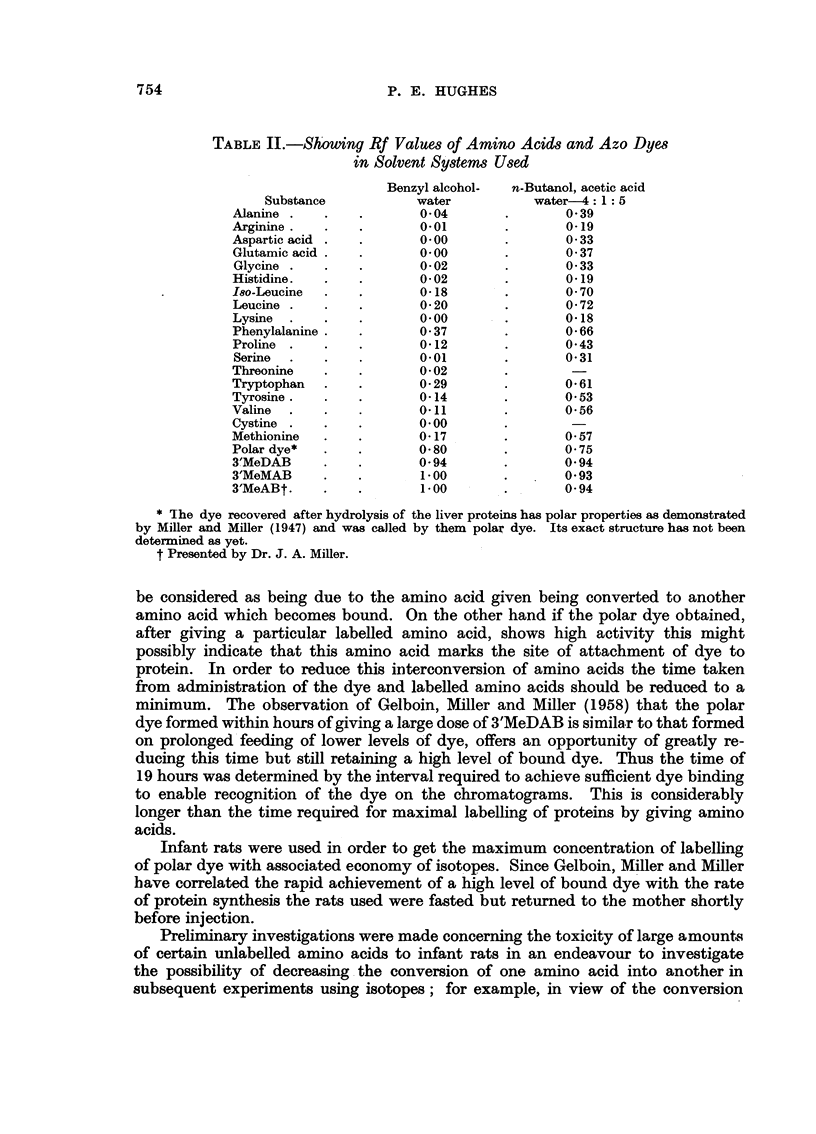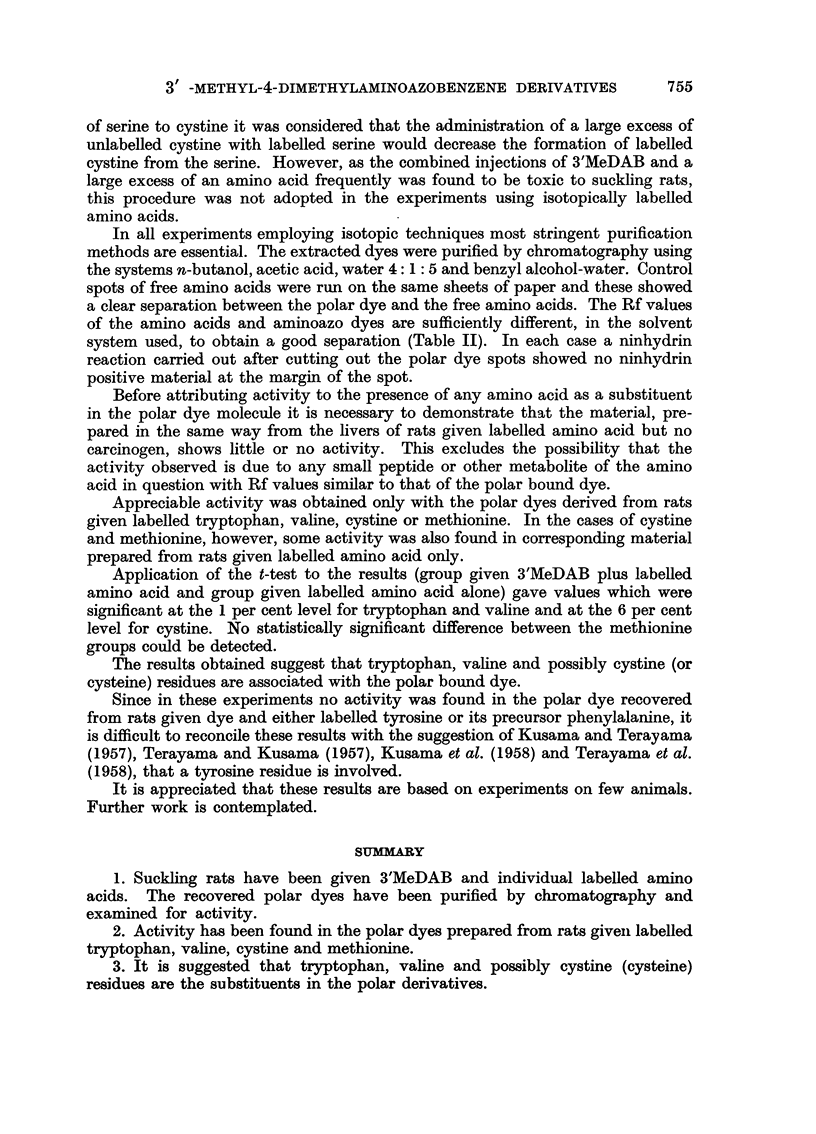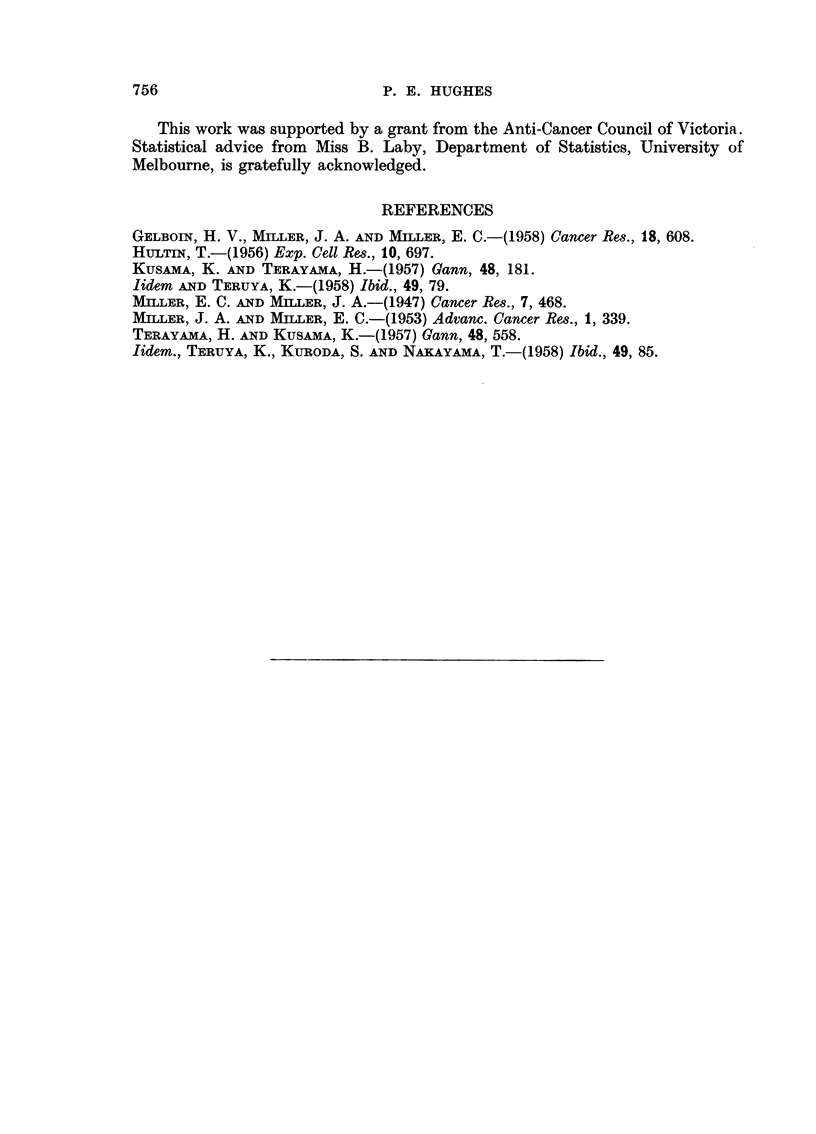# Concerning the Origin of Substituents in Polar-Bound Derivatives of 3′-Methyl-4-Dimethylaminoazobenzene

**DOI:** 10.1038/bjc.1959.84

**Published:** 1959-12

**Authors:** P. E. Hughes


### CONCERNING THE ORIGIN OF SUBSTITUENTS IN POLAR-BOUND DERIVATIVES OF 3′-METHYL-4-DIMETHYLAMINOAZOBENZENE

P. E. HUGHES

*From the Department of Pathology, University of Melbourne, Melbourne, Australia*

Received for publication October 23, 1959

FOLLOWING the demonstration by Miller and Miller (1947) of the binding of carcinogenic aminoazo dyes to proteins of the target organ, much work has been directed towards elucidating the reactive sites of both carcinogen and protein.

Miller and Miller (1953) have suggested that the linkage between dye and protein is stable to treatment with hot ethanolic KOH. Under these conditions hydrolysis of protein occurs so that the recovered polar dye should have an amino acid residue attached. To date, insufficient quantities of this polar derivative have been obtained to enable chemical identification of this residue.

Gelboin, Miller and Miller (1958) have reported that, a few hours after the intraperitoneal administration of a large dose of 3′-methyl-4-dimethylaminoazobenzene (3′MeDAB), protein-bound derivatives are detectable in the liver. The electrophoretic and spectral properties of the bound dyes obtained, either by prolonged feeding or following a single dose, were similar. The findings of Gelboin, Miller and Miller (1958) support the view that a large percentage of the bound dye is bound to certain liver proteins during their synthesis rather than by becoming attached to protein already formed. This interpretation is in disagreement with that of Hultin (1956) who suggested that the formation of protein-bound dye is independent of protein synthesis.

To date, little evidence is available concerning the actual site(s) involved in the protein(s) concerned. Kusama and Terayama (1957), Terayama and Kusama (1957), Kusama, Terayama and Teruya (1958) and Terayama, Kusama, Teruya, Kuroda and Nakayama (1958), as a result of spectroscopic studies of synthetic polar-like dyes, have suggested that a tyrosine-aminoazobenzene combination is the most probable model for the polar dye.

In this communication the results of an investigation, using isotopically labelled amino acids in an endeavour to determine the origin of substituents in the polar derivative(s) of aminoazo dyes, are presented.

### METHODS

### *Care of animals*

As a locally available strain of Wistar rats had previously been found to give higher levels of protein-bound dye than rats of Sprague-Dawley strain, the former were used.

Three of four rats, weighing 18–31 g. were removed from their mother and kept warm for five hours. They were then injected intraperitoneally with 0·5 ml. of a

2 per cent solution of 3′MeDAB in olive oil and returned to the mother after the remaining members of the litter had been removed. After a further hour the young rats were inspected and ones selected were injected intraperitoneally with $^{14}C$ labelled amino acid.* A dose of 0·01 mc of amino acid was given when the L isomer was used while 0·020–0·025 mc was given when DL amino acids were employed. The following amino acids were used : DL Alanine-1-$^{14}C$, L Arginine-$^{14}C$ (G), L Aspartic acid-$^{14}C$ (G), L Glutamic acid-$^{14}C$ (G), Glycine-1-$^{14}C$, L Histidine-2(ring)-$^{14}C$, *Iso*-Leucine-$^{14}C$ (G), DL Leucine-1-$^{14}C$, L Lysine-$^{14}C$ (G), L Phenylalanine-$^{14}C$ (G), L Proline-$^{14}C$ (G), L Serine-3-$^{14}C$, L Serine-$^{14}C$ (G), L Threonine-$^{14}C$ (G), DL Tryptophan (Indolyl-Alanine-3-$^{14}C$), L Tyrosine-$^{14}C$ (G), DL Valine-1-$^{14}C$, DL Cystine-$^{35}S$ and DL Methionine-$^{35}S$. After a further period of 19 hours the rats were sacrificed and their livers removed and homogenized in 2 ml. of water.

*Preparation of polar dyes, chromatography and counting techniques*

After adding an aliquot of 0·5 M pH 4·0 acetate buffer the liver proteins were precipitated by boiling for three minutes. The precipitated proteins were collected on hardened filter paper, washed with ethanol and then extracted for 48 hours with ethanol in a Soxhlet apparatus. The extracted powders were then digested in ethanolic KOH at 80° C. for 20 hours and the digests extracted twice with 1 : 5 ethanol-ethyl ether by the method of Miller and Miller (1947). The combined ethereal extracts were transferred to small beakers. KOH dissolved in the ethereal extracts was precipitated as carbonate by the addition of a small piece of dry ice and the extract was evaporated to dryness, initially over a water bath and finally *in vacuo*. The residues (apart from potassium carbonate) were transferred by dissolving in 1 : 5 ethanol-ethyl ether to 2·2 cm. diameter watch glasses and any activity determined by using a Geiger Muller tube with a mica window 2·3 mg. cm.$^{-2}$ ; counting was performed at infinite thinness for a minimum time of 30 minutes for each sample (background 7–10 counts per minute).

The samples were transferred to Whatman No. 1 paper with a small amount of ethanol. The total amount of polar dye extracted from one liver was introduced on to the paper in three spots. If necessary additional unlabelled polar dye eluted from other chromatograms was added to make the spots visible. The spots were dried with a hair drier between each application and then chromatographed using the solvent system $n$-butanol, acetic acid, water 4 : 1 : 5. Prior to chromatography the spots were exposed to ammonia vapour. After chromatography and drying, the papers were exposed to HCl vapour and any areas showing a faint pink colour were cut out and eluted with ethanol in a semi-micro Soxhlet apparatus. After standing under a hood for 3–4 hours the remainder of the paper was sprayed with ninhydrin. After drying *in vacuo* the eluted material from the pink spots was then rechromatographed on Whatman No. 1 paper using the system benzyl alcohol, water and once again the areas showing a faint pink colour on acidification were cut out and eluted with ethanol. All the polar dye arising from a single liver (which was run in three spots on the initial chromatograms) was then pooled and run as a single spot in the benzyl alcohol, water system. After drying, the diluted material was transferred to watch glasses and counted as before at infinite thinness, for a minimum time of 30 minutes. The density of material on the sources counted was not greater than 10 $\mu$g. cm.$^{-2}$ The material counted in each case was

* Obtained from the Radiochemical Centre, Amersham, Bucks., England.

thus the total amount of polar dye derived from a single liver. Counting was performed for at least half an hour with each source ; background varied between 8 and 10 counts per minute. The amount of azo dye present in each source was estimated spectrophotometrically in HCl-ethanol at 520 m$\mu$. A solution of 3′MeDAB in acid-ethanol was used as a standard.

In all cases control spots of known unlabelled amino acid were run on the same piece of paper with the dye-labelled amino acid samples. As a result of a preliminary screening experiment tryptophan, valine, histidine and proline were subjected to further study. Using these amino acids, methionine and cystine, litter mates were given either a labelled amino acid alone or a labelled amino acid plus carcinogen and their livers subsequently hydrolyzed and extracted in an identical manner. Both samples were then chromatographed on the same sheet of paper and corresponding areas were cut out, eluted and counted.

All chromatography was performed at 23 $\pm$ 2° C.

### RESULTS

The polar derivatives of 3′MeDAB prepared from livers of rats given $^{14}C$ labelled alanine arginine, aspartic acid, glutamic acid, glycine, histidine, *iso*-leucine, leucine, lysine, phenylalanine, proline, serine, threonine and tyrosine showed little or no activity. All of these experiments were run at least in duplicate ; in some cases the procedure was carried out four times.

The polar dyes obtained from rats given labelled tryptophan, valine, methionine and cystine showed appreciable activity (Table I). While material prepared in the same way from rats given labelled tryptophan only (without 3′MeDAB) or labelled valine only showed very low activity, that prepared from rats given $^{35}S$ labelled methionine or cystine only showed some activity.

Rf values of amino acids and aminoazo dyes are shown in Table II.

TABLE I.—*Showing Activities of Polar Dyes Obtained from Rats given Labelled Tryptophan, Valine, Cystine and Methionine*

| Amino acid | Number of rats | Mean counts per minute per mg. DF polar dye | Level of statistical significance of difference between group given carcinogen and control group (*t*-test) |
|---|---|---|---|
| Tryptophan | 8 | 4495±285* | <1% |
| Valine | 7 | 3268±252* | <1% |
| Cystine | 4 | 2887±356* | 6% |
| Methionine | 4 | 4420±962 | Not significant |

* = Standard deviation.

### DISCUSSION

Interpretations drawn from studies of this type must be viewed within the limitations of the technique. The metabolic interconversion of amino acids is well established so that if the polar derivative of 3′MeDAB prepared from rats given a particular $^{14}C$ labelled amino acid shows a significant but low activity, this should

TABLE II.—*Showing Rf Values of Amino Acids and Azo Dyes in Solvent Systems Used*

| Substance | Benzyl alcohol-water | *n*-Butanol, acetic acid water—4 : 1 : 5 |
|---|---|---|
| Alanine | 0·04 | 0·39 |
| Arginine | 0·01 | 0·19 |
| Aspartic acid | 0·00 | 0·33 |
| Glutamic acid | 0·00 | 0·37 |
| Glycine | 0·02 | 0·33 |
| Histidine | 0·02 | 0·19 |
| *Iso*-Leucine | 0·18 | 0·70 |
| Leucine | 0·20 | 0·72 |
| Lysine | 0·00 | 0·18 |
| Phenylalanine | 0·37 | 0·66 |
| Proline | 0·12 | 0·43 |
| Serine | 0·01 | 0·31 |
| Threonine | 0·02 | — |
| Tryptophan | 0·29 | 0·61 |
| Tyrosine | 0·14 | 0·53 |
| Valine | 0·11 | 0·56 |
| Cystine | 0·00 | — |
| Methionine | 0·17 | 0·57 |
| Polar dye* | 0·80 | 0·75 |
| 3'MeDAB | 0·94 | 0·94 |
| 3'MeMAB | 1·00 | 0·93 |
| 3'MeAB† | 1·00 | 0·94 |

* The dye recovered after hydrolysis of the liver proteins has polar properties as demonstrated by Miller and Miller (1947) and was called by them polar dye. Its exact structure has not been determined as yet.

† Presented by Dr. J. A. Miller.

be considered as being due to the amino acid given being converted to another amino acid which becomes bound. On the other hand if the polar dye obtained, after giving a particular labelled amino acid, shows high activity this might possibly indicate that this amino acid marks the site of attachment of dye to protein. In order to reduce this interconversion of amino acids the time taken from administration of the dye and labelled amino acids should be reduced to a minimum. The observation of Gelboin, Miller and Miller (1958) that the polar dye formed within hours of giving a large dose of 3'MeDAB is similar to that formed on prolonged feeding of lower levels of dye, offers an opportunity of greatly reducing this time but still retaining a high level of bound dye. Thus the time of 19 hours was determined by the interval required to achieve sufficient dye binding to enable recognition of the dye on the chromatograms. This is considerably longer than the time required for maximal labelling of proteins by giving amino acids.

Infant rats were used in order to get the maximum concentration of labelling of polar dye with associated economy of isotopes. Since Gelboin, Miller and Miller have correlated the rapid achievement of a high level of bound dye with the rate of protein synthesis the rats used were fasted but returned to the mother shortly before injection.

Preliminary investigations were made concerning the toxicity of large amounts of certain unlabelled amino acids to infant rats in an endeavour to investigate the possibility of decreasing the conversion of one amino acid into another in subsequent experiments using isotopes ; for example, in view of the conversion of serine to cystine it was considered that the administration of a large excess of unlabelled cystine with labelled serine would decrease the formation of labelled cystine from the serine. However, as the combined injections of 3′MeDAB and a large excess of an amino acid frequently was found to be toxic to suckling rats, this procedure was not adopted in the experiments using isotopically labelled amino acids.

In all experiments employing isotopic techniques most stringent purification methods are essential. The extracted dyes were purified by chromatography using the systems *n*-butanol, acetic acid, water 4 : 1 : 5 and benzyl alcohol-water. Control spots of free amino acids were run on the same sheets of paper and these showed a clear separation between the polar dye and the free amino acids. The Rf values of the amino acids and aminoazo dyes are sufficiently different, in the solvent system used, to obtain a good separation (Table II). In each case a ninhydrin reaction carried out after cutting out the polar dye spots showed no ninhydrin positive material at the margin of the spot.

Before attributing activity to the presence of any amino acid as a substituent in the polar dye molecule it is necessary to demonstrate that the material, prepared in the same way from the livers of rats given labelled amino acid but no carcinogen, shows little or no activity. This excludes the possibility that the activity observed is due to any small peptide or other metabolite of the amino acid in question with Rf values similar to that of the polar bound dye.

Appreciable activity was obtained only with the polar dyes derived from rats given labelled tryptophan, valine, cystine or methionine. In the cases of cystine and methionine, however, some activity was also found in corresponding material prepared from rats given labelled amino acid only.

Application of the *t*-test to the results (group given 3′MeDAB plus labelled amino acid and group given labelled amino acid alone) gave values which were significant at the 1 per cent level for tryptophan and valine and at the 6 per cent level for cystine. No statistically significant difference between the methionine groups could be detected.

The results obtained suggest that tryptophan, valine and possibly cystine (or cysteine) residues are associated with the polar bound dye.

Since in these experiments no activity was found in the polar dye recovered from rats given dye and either labelled tyrosine or its precursor phenylalanine, it is difficult to reconcile these results with the suggestion of Kusama and Terayama (1957), Terayama and Kusama (1957), Kusama *et al.* (1958) and Terayama *et al.* (1958), that a tyrosine residue is involved.

It is appreciated that these results are based on experiments on few animals. Further work is contemplated.

### SUMMARY

1. Suckling rats have been given 3′MeDAB and individual labelled amino acids. The recovered polar dyes have been purified by chromatography and examined for activity.

2. Activity has been found in the polar dyes prepared from rats given labelled tryptophan, valine, cystine and methionine.

3. It is suggested that tryptophan, valine and possibly cystine (cysteine) residues are the substituents in the polar derivatives.

This work was supported by a grant from the Anti-Cancer Council of Victoria. Statistical advice from Miss B. Laby, Department of Statistics, University of Melbourne, is gratefully acknowledged.

### REFERENCES

GELBOIN, H. V., MILLER, J. A. AND MILLER, E. C.—(1958) *Cancer Res.*, **18**, 608.
HULTIN, T.—(1956) *Exp. Cell Res.*, **10**, 697.
KUSAMA, K. AND TERAYAMA, H.—(1957) *Gann*, **48**, 181.
*Iidem* AND TERUYA, K.—(1958) *Ibid.*, **49**, 79.
MILLER, E. C. AND MILLER, J. A.—(1947) *Cancer Res.*, **7**, 468.
MILLER, J. A. AND MILLER, E. C.—(1953) *Advanc. Cancer Res.*, **1**, 339.
TERAYAMA, H. AND KUSAMA, K.—(1957) *Gann*, **48**, 558.
*Iidem.*, TERUYA, K., KURODA, S. AND NAKAYAMA, T.—(1958) *Ibid.*, **49**, 85.